# Cumulative caffeine exposure predicts neurodevelopmental outcomes in premature infants

**DOI:** 10.1038/s41390-025-04387-1

**Published:** 2025-09-20

**Authors:** Bridget E. L. Ostrem, Elizabeth Odell, Kimberly N. Grelli, Katelin Kramer, Natalie Chan, Fei Jiang, Duan Xu, A. James Barkovich, Matthew J. Barkovich, Donna M. Ferriero, Dawn Gano

**Affiliations:** 1 Weill Institute for Neurosciences, Department of Neurology, University of California, San Francisco, San Francisco, CA USA; 2 Divison of Neonatology, UCSF Benioff Children’s Hospital Oakland, Oakland, CA USA; 3 Department of Pediatrics, University of California, San Francisco, San Francisco, CA USA; 4 Department of Epidemiology and Biostatistics, University of California, San Francisco, San Francisco, CA USA; 5 Department of Radiology and Biomedical Imaging, University of California, San Francisco, San Francisco, CA USA; 6 Department of Pediatrics, University of British Columbia, BC Children’s Hospital Research Institute, Vancouver, Canada

## Abstract

**Background:**

Preterm infants who receive caffeine for apnea of prematurity have improved neurodevelopmental outcomes compared to untreated infants. The optimal dosing regimen for neuroprotection is unknown. We hypothesized that higher caffeine exposure is associated with improved neurodevelopmental performance.

**Methods:**

We quantified caffeine exposure in a previously reported cohort of 106 infants born at ≤32 gestational weeks who received brain MRIs during the neonatal hospitalization. Infants were subdivided into tertiles based on average daily caffeine exposure (ADCE). Bayley-III examinations were performed on 69 participants at 30 months corrected age. Neurodevelopmental impairment (NDI) was defined as a score of ≤85 on the Bayley-III motor, language, and/or cognitive subscales. We evaluated the relationship between caffeine exposure, neuroimaging abnormalities, and neurodevelopmental performance.

**Results:**

Higher ADCE was associated with decreased odds of NDI (OR 0.69, 95% C.I. 0.50–0.95) but not with MRI abnormalities. High dose caffeine was associated with improved motor (mean difference 10.9, 95% C.I. 0.7–21.0), language (mean difference 15.2, 95% C.I. 3.4–27.0), and cognitive (mean difference 13.0, 95% C.I. 0.6–25.4) performance compared to low dose in multivariable analyses adjusted for gestational age and respiratory disease.

**Conclusion:**

Higher sustained caffeine exposure during the neonatal hospitalization is associated with improved neurodevelopmental outcomes in preterm infants.

**Impact:**

- Premature infants who receive caffeine for apnea of prematurity have improved neurodevelopmental outcomes compared to untreated infants.
- The optimal dosing strategy for neuroprotection is unknown.
- We found that higher average daily exposure during the neonatal hospitalization was associated with reduced neurodevelopmental impairment at 30 months corrected age.
- High dose caffeine was associated with improved motor, language, and cognitive performance on the Bayley-III compared to low dose caffeine in multivariable analyses adjusted for gestational age and respiratory disease.
- Preterm infants may benefit from higher maintenance doses and/or from continuing caffeine beyond the period of respiratory need.

## Introduction

Advancements in neonatal intensive care have led to improved survival rates for preterm infants in the U.S and around the world.^1,2^ However, preterm infants experience higher rates of brain injury than full-term infants, and are at increased risk for long-term disabilities, including motor, cognitive, vision, hearing, attention, social-behavioral, and learning impairments.^3–7^ The incidence of acquired brain injury, including white matter injury (WMI) and germinal matrix hemorrhage/intraventricular hemorrhage (GMH/IVH), increases with decreasing gestational age at birth.^5^ Brain injury is most common in babies born before 32 weeks of completed gestation. There are few treatments available postnatally to prevent preterm brain injury, promote brain recovery after injury, or improve neurodevelopmental outcomes in infants at high risk for future impairment.^5^

Caffeine, a central nervous system stimulant and methylxanthine that antagonizes adenosine-mediated suppression of respiratory drive, is widely administered to preterm neonates with apnea of prematurity.^8^ Caffeine administration reduces the incidence of bronchopulmonary dysplasia (BPD) and decreases the need for mechanical ventilation.^9,10^ The long-term neurodevelopmental benefits of caffeine were identified after analysis of follow-up data from the Caffeine for Apnea of Prematurity (CAP) Trial.^9^ Preterm neonates with birth weights of 500–1250 g were randomized to receive placebo or caffeine, with a loading dose of 20 mg/kg followed by maintenance dosing of 5–10 mg/kg/day until respiratory status improved. Participants who received caffeine had a lower risk of motor impairment at 18 months corrected age, improved fine motor coordination at 5 years, and enhanced visuomotor, visuoperceptual, and visuospatial abilities at 11 years old.^11–14^

Despite multiple follow-up studies, it is unknown whether an alternative maintenance dosing caffeine strategy would maximize the neuroprotective effects of caffeine, alter rates of neurological injury, or improve long-term outcomes in preterm infants.^10,15^ Animal models suggest that sustained daily caffeine exposure throughout preterm brain development promotes myelination and recovery from WMI.^16,17^ We sought to determine the association between cumulative and daily caffeine exposure over the neonatal hospitalization with neuroimaging abnormalities and neurodevelopmental outcomes in a prospective cohort of preterm infants born at equal to or less than 32 weeks’ gestation. We hypothesized that higher caffeine exposure would be associated with a lower risk of acquired brain injury and a lower risk of neurodevelopmental impairment.

## Methods

### Study design and setting

This study was a retrospective secondary analysis of infants prospectively enrolled in the Prematurely Born Neonate MRI (PREMRI) study, admitted to the University of California, San Francisco (UCSF) Benioff Children’s Hospital Intensive Care Nursery between 2011 and 2016. Preterm infants less than 32 weeks at birth were eligible for enrollment except if one or more of the following exclusion criteria were present: congenital malformation, genetic syndrome, congenital infection, or instability for transport to MRI. Infants enrolled in the PREMRI cohort had up to two brain MRIs performed during the neonatal hospitalization. For this study, we analyzed the results of the MRI scan performed closest to term-equivalent age. Of the 108 infants enrolled over the study period, we excluded 2 infants with no MRI scans in our analysis of caffeine dosing parameters and brain MRI abnormalities. Neurodevelopmental follow-up data through 30 months corrected age (CA) were available in 69 participants. The UCSF Institutional Review Board approved this study, and parental consent for study participation was obtained.

### Caffeine dosing parameters

Information regarding caffeine dose, timing, route of administration, and weights during the neonatal hospitalization for each subject was retrospectively drawn from the electronic health record (EHR). For each hospital day in which caffeine was administered, the dose in milligrams (mg) was divided by the most recent daily weight in kilograms (kg) to obtain the day’s weight-based caffeine dose in mg per kg (mg/kg). Measures of cumulative caffeine dosing were calculated as follows: The average maintenance dose was calculated as the sum of the daily doses of caffeine (excluding the bolus dose typically given on the first day of dosing) divided by the length of the caffeine treatment course. The daily weight-based doses, including the bolus dose and all maintenance doses, were summed to obtain the cumulative caffeine exposure (CCE) for each subject. CCE was divided by the number of days a subject was born before the due date to obtain the average daily caffeine exposure (ADCE).

### Clinical parameters

Neonatal demographic and clinical variables, including gestational age (GA) at birth, birth weight, sex, prenatal steroid administration, maternal chorioamnionitis, surfactant administration, infection, patent ductus arteriosus (PDA), necrotizing enterocolitis (NEC), and neonatal surgery, were collected prospectively by a clinical research nurse as previously described.^18^ Bell stage II criteria or higher were used to diagnose NEC.^19^ BPD was classified utilizing the NIH consensus criteria, based on respiratory support and fraction of inspired oxygen (FiO_2_) required at 36 weeks postmenstrual age (PMA).^20^ Use of nasal cannula at any flow was classified as moderate BPD. Cumulative supplemental oxygen in the first two weeks (CSO_1–14_) and first four weeks (CSO_1–28_) of life was calculated as previously described.^18^ Briefly, CSO was calculated as a daily average of supplemental oxygen (recorded FiO_2_ – 0.21) to estimate the overall supplemental oxygen exposure on a given day, and was then summed over the appropriate time period.^21^ Utilizing the STOP-ROP guidelines, the recorded FiO_2_ was converted to an effective FiO_2_ when the infant was on nasal cannula.^22^

### Magnetic resonance imaging

MRI scans were acquired using a 3T-scanner (General Electric Discovery MR750; GE Medical Systems, Waukesha, Wisconsin) and included axial fast spin-echo T2-weighted images (repetition time, 5000 ms; echo time, 120 ms; field of view, 20 cm with 256 × 256 matrix; slice thickness, 2 mm; gap, 0 mm), sagittal volumetric 3-dimensional spoiled gradient echo T1-weighted images (inversion time, 450 ms; echo time, minimal; field of view, 18 cm; 1.0 mm isotropic), and susceptibility weighted imaging (SWI) (TE, minimal; TR, 25 ms; FOV, 18 cm; slice thickness, 2.2 mm). Presence and severity of WMI and GMH/IVH were scored based on the Miller and Papile grading systems, respectively, by a blinded pediatric neuroradiologist as previously described.^23,24^ The presence of cerebellar hemorrhage was determined by review of T2-weighted and SWI sequences.

### Neurodevelopmental assessments

After hospital discharge, infants were referred to the UCSF Intensive Care Nursery Follow-Up Program for routine follow-up. Neurodevelopment was assessed using the Bayley Scales of Infant and Toddler Development, 3rd edition (Bayley-III), which was performed at 30 months CA by clinicians who were not blinded to the medical history.^25^ Follow-up was available in 69 subjects. The primary outcome assessed in this study was a composite outcome of neurodevelopmental impairment (NDI) at 30 months CA, defined as a composite score of 85 or lower on the motor, language, and/or cognitive subscales of the Bayley-III.

### Statistical analyses

Statistical analyses were performed using Stata version 17 (College Station, Texas) except where noted below. Where subjects did not have data available regarding a particular exposure or outcome of interest, we excluded those subjects from the relevant analysis. For all statistical tests, a *p* value of less than 0.05 was considered significant.

Shapiro–Wilk tests were used to assess for normality of each variable and Brown-Forsythe tests were used to assess for equal variance prior to comparisons between groups. We reported descriptive statistics with mean and standard deviation (SD), median and interquartile range (IQR), or frequency, as appropriate. Clinical characteristics were assessed in the cohort as a whole and compared between newborns with NDI versus no NDI at 30 months CA using Fisher exact tests for categorical variables, Student’s *t*-tests for parametric continuous variables, and Wilcoxon Rank-Sum tests for ordinal and non-parametric continuous variables. The association of caffeine dosing parameters with the binary outcome of NDI was evaluated using logistic regression. The association of caffeine dosing parameters with the presence of each neuroimaging abnormality was evaluated using logistic regression.

To further investigate outcomes dependent on caffeine dose level, the 69 subjects with developmental follow-up were ranked by ADCE and subdivided into exact tertiles (23 subjects/group). Clinical and caffeine dosing characteristics were compared between the high, mid, and low-dose groups using GraphPad Prism version 10. *P* values were adjusted for multiple comparisons. Normally distributed continuous variables with equal variance were compared using one-way ANOVA followed by Tukey’s post hoc tests for pairwise comparisons. Continuous variables that were not normally distributed were compared using a Kruskal–Wallis test with post hoc Dunn’s tests. Continuous variables that were normally distributed and had unequal variance were compared using a Welch’s one-way ANOVA followed by Dunnett post hoc tests. Categorical variables were compared with Fisher’s exact tests followed by Bonferroni correction for multiple comparisons. Linear regression was used to evaluate the relationship between caffeine dose group and motor, language, and cognitive scores on the Bayley-III in univariate and multivariable models. We confirmed multivariable linear regression model assumptions for normality and linearity. Mean difference between the high and low dose groups corresponds to the effect of an approximately 3.9 mg/kg higher average daily caffeine exposure.

## Results

### Clinical characteristics of the patient cohort

In this cohort of 106 subjects, the mean (SD) GA at birth was 27.7 (±2.2) weeks and the median birthweight was 980 (IQR 750, 1255) grams (Table 1). Ninety-seven subjects (92%) received caffeine, with a median loading dose of 20.2 mg/kg (IQR 20.0, 21.2). Caffeine dosing was initiated at a median age of 1 day old (IQR 0, 5). Neurodevelopmental follow-up was available in 69 subjects at a median age of 29.8 (IQR 21.0, 31.6) months corrected age (CA). Patients with neurodevelopmental follow-up did not differ significantly in baseline characteristics or neuroimaging findings compared to subjects without follow-up (Supplementary Table S1).

**Table 1:** Characteristics of the cohort.

Neonatal characteristics	Study Cohort (*N* = 106)	No NDI (*N* = 46)	NDI (*N* = 23)	*p* value^a^
GA at birth, mean wks (SD)	27.7 (2.2)	27.5 (2.0)	27.4 (2.6)	0.906
Birthweight, median grams (IQR)	980 (750, 1255)	990 (760, 1190)	1030 (680, 1160)	0.376
Male, *n* (%)	54 (51)	23 (50)	12 (52)	1.000
Prenatal steroids, *n* (%)	82 (77)	36 (78)	18 (78)	1.000
Maternal chorioamnionitis, *n* (%)	11 (10)	6 (13)	1 (4)	0.411
PDA, *n* (%)	60 (57)	31 (67)	13 (57)	0.432
Necrotizing enterocolitis, *n* (%)	6 (6)	2 (4)	3 (13)	0.324
BPD, *n* (%)	61 (57)	32 (70)	11 (48)	0.114
CSO_1–14_, median (IQR)	0.32 (0.03, 0.83)	0.39 (0.08, 0.88)	0.30 (0, 1.39)	0.660
CSO_1–28_, median (IQR)	0.57 (0.07, 1.80)	0.73 (0.12, 2.28)	0.32 (0, 3.47)	0.751
Received caffeine, *n* (%)	97 (92)	44 (96)	20 (87)	0.324
Age at first caffeine dose, median days (IQR)	1 (0,5)	2 (0, 6)	1 (0, 7.5)	0.912
Loading caffeine dose, median mg/kg (IQR)	20.2 (20.0, 21.2)	20.1 (20.0, 20.8)	20.4 (20.0, 21.5)	0.468
Maintenance dose, median mg/kg (IQR)	7.6 (6.3, 8.7)	7.8 (6.5, 8.9)	7.1 (4.9, 8.6)	0.220
Average daily caffeine exposure (ADCE), mean mg/kg (SD)	3.3 (1.9)	3.7 (1.8)	2.7 (1.5)	0.017
Cumulative caffeine exposure (CCE), mean mg/kg (SD)	301.3 (196.4)	339.5 (27.8)	249.1 (35.6)	0.057
Postmenstrual age at MRI, median weeks (IQR)	36.1 (34.9, 37.9)	35.4 (34.0, 36.9)	36.9 (35.9, 38.1)	0.029
WMI on MRI, available *N*	*N* = 89	*N* = 38	*N* = 21	0.625
None, *n* (%)	64 (72)	29 (76)	15 (71)	
Mild, *n* (%)	16 (18)	7 (18)	4 (19)	
Moderate, *n* (%)	5 (6)	2 (5)	1 (5)	
Severe, *n* (%)	4 (4)	0 (0)	1 (5)	
GMH/IVH on MRI, available *N*	*N* = 101	*N* = 45	*N* = 23	0.718
None, *n* (%)	69 (68)	31 (69)	15 (65)	
Grade 1, *n* (%)	8 (8)	3 (7)	2 (9)	
Grade 2, *n* (%)	16 (16)	9 (20)	4 (17)	
Grade 3, *n* (%)	4 (4)	2 (4)	1 (4)	
PVHI, *n* (%)	4 (4)	0 (0)	1 (4)	
Cerebellar hemorrhage, n out of available *N* (%)	30/101 (30)	16/45 (36)	7/23 (30)	0.789
Corrected age at follow-up, median months (IQR)	29.8 (21.0, 31.6)	29.0 (18.9, 30.8)	31.8 (27.3, 32.9)	0.002

Available *N* are shown for neuroimaging findings, as motion artifact precluded full characterization of all MRI features in all scans.

*SD* standard deviation, *IQR* interquartile range. *WMI* white matter injury severity on last brain MRI scan during the neonatal hospitalization, *GMH/IVH* germinal matrix hemorrhage/ intraventricular hemorrhage severity on last brain MRI scan during the neonatal hospitalization, *PVHI* periventricular hemorrhagic infarction.

^a^Comparisons were made between no NDI and NDI groups using Fisher exact tests for categorical variables, Student’s *t*-tests for parametric continuous variables, and Wilcoxon Rank-Sum tests for ordinal and non-parametric continuous variables.

We compared clinical characteristics and neuroimaging findings in subjects with and without evidence of neurodevelopmental impairment (NDI), defined as a composite score of 85 or lower on the motor, language, and/or cognitive subscale of the Bayley-III (Table 1). The median postmenstrual age at MRI was older in the NDI (36.9 weeks) group compared to the no NDI (35.4 weeks) group (*p* = 0.029, Wilcoxon Rank-Sum test). The median corrected age at last follow-up was older in the NDI group (31.8 months) as compared to the no NDI group (29.0 months). Subjects with NDI did not differ significantly from subjects without NDI with regards to prenatal exposures, GA at birth, neonatal comorbidities, or frequency of brain MRI abnormalities.

### Caffeine association with neuroimaging abnormalities

We asked whether caffeine dosing characteristics were associated with acquired brain injury on neonatal MRI. Caffeine dosing variables included the average maintenance dose (sum of the daily weight-based doses, excluding the bolus dose, divided by the length of the caffeine treatment course), cumulative caffeine exposure (CCE), which is the sum of all caffeine doses given during the neonatal hospitalization, and the average daily caffeine exposure (ADCE), which is the CCE was divided by the number of days a subject was born before the due date, as an estimation of the hospitalization length and eligible window during which caffeine could possibly be given (Supplementary Fig. S1). Using univariate logistic regression, higher maintenance caffeine dose, CCE, and ADCE were all associated with higher odds of moderate or severe WMI (Table 2). However, we also found that BPD severity and PDA were associated with higher odds of moderate or severe WMI in univariate models. Using multivariable logistic regression controlling for BPD severity and PDA as covariates, we no longer observed an association between maintenance caffeine dose, CCE, ADCE, or PDA, and moderate to severe WMI. BPD severity remained associated with increased odds of moderate or severe WMI in the multivariable analysis (adjusted odds ratio [aOR] 1.79, 95% confidence interval [C.I.] 1.08, 2.96, p = 0.023). Thus, increased BPD severity in patients who received higher caffeine exposures likely explained the apparent association between caffeine exposure and WMI in our initial univariate analysis. We further found that caffeine dosing parameters were not associated with risk of severe GMH/IVH (grade 3 or periventricular hemorrhagic infarction [PVHI]) or cerebellar hemorrhage (Table 2). BPD severity was associated with increased odds of cerebellar hemorrhage in both univariate and multivariable models (aOR 1.67, 95% C.I. 1.01, 2.77, *p* = 0.047). These observations support prior studies reporting BPD as a risk factor for both WMI^18^ and cerebellar hemorrhage.^26^

**Table 2:** Association of caffeine and neonatal comorbidities with MRI abnormalities.

Predictor	Moderate or severe WMI			Grade 3 GMH/IVH or PVHI			Cerebellar hemorrhage		
	OR	95% CI	*p* value	OR	95% CI	*p* value	OR	95% CI	*p* value
Univariate									
Maint. dose	1.45	1.06, 1.97	0.019	1.13	0.76, 1.66	0.549	1.01	0.77, 1.33	0.950
ADCE	1.50	1.14, 1.97	0.004	0.97	0.71, 1.33	0.863	1.03	0.81, 1.29	0.823
CCE	1.00	1.00, 1.01	0.004	1.00	1.00, 1.00	0.967	1.00	1.00, 1.00	0.470
BPD severity	2.20	1.39, 3.48	0.001	1.56	0.90, 2.69	0.111	1.57	1.03, 2.40	0.036
PDA	3.33	1.21, 9.17	0.020	1.85	0.53, 6.45	0.332	1.50	0.62, 3.60	0.365
GA at birth	0.85	0.69, 1.05	0.125	1.05	0.81, 1.37	0.717	0.84	0.69, 1.03	0.100
Multivariable									
Maint. dose^a^	1.28	0.93, 1.77	0.124	0.99	0.66, 1.48	0.962	0.90	0.66, 1.23	0.515
ADCE^a^	1.28	0.95, 1.71	0.100	0.83	0.59, 1.18	0.307	0.96	0.73, 1.25	0.742
CCE^a^	1.00	1.00, 1.00	0.181	1.00	0.99, 1.00	0.323	1.00	1.00, 1.00	0.946
BPD severity^b^	1.79	1.08, 2.96	0.023	1.61	0.88, 2.97	0.124	1.67	1.01, 2.77	0.047
PDA^b^	2.05	0.69, 6.14	0.199	1.56	0.39, 6.18	0.528	0.87	0.32, 2.42	0.795

*PVHI* periventricular hemorrhagic infarction. *Maint, dose* maintenance dose, *ADCE* average daily caffeine exposure, *CCE* cumulative caffeine exposure, *BPD* bronchopulmonary dysplasia, *PDA* patent ductus arteriosus, *GA* gestational age at birth.

^a^Multivariable model included the caffeine dosing parameter of interest, BPD severity, and PDA as covariates.

^b^Multivariable model included BPD severity, PDA, and ADCE as covariates.

### Caffeine exposure is associated with improved neurodevelopmental performance

The proportion of subjects who received caffeine, the age at first caffeine dose, the loading dose, the maintenance dose of caffeine, and CCE did not differ significantly between subjects with and without NDI (Table 1). ADCE was higher in the patients with no NDI (3.7 ± 1.8 mg/kg) as compared to the patients with NDI (2.7 ± 1.5 mg/kg) (*p* = 0.017, Student’s *t*-test). We used logistic regression to further explore the association between caffeine exposure and NDI (Table 3). Age at first caffeine dose and maintenance dose were not significantly associated with NDI in univariate models, or in multivariable models that included GA at birth and corrected age at follow-up as covariates. Higher ADCE was associated with significantly lower odds of NDI in univariate (odds ratio [OR] 0.69, 95% C.I. 0.50, 0.95, *p* = 0.016) and in multivariable (aOR 0.58, 95% C.I. 0.39, 0.87, *p* = 0.009) analyses. CCE was also associated with lower odds of NDI in multivariable (aOR 0.99, 95% C.I. 0.99, 1.00, *p* = 0.010) but not univariate analysis. Based on these results, for every 10 mg/kg increase in CCE, there is a 10% reduction in the odds of NDI, independent of GA at birth and corrected age at follow-up. Severity of WMI and severity of GMH/IVH were not significantly associated with NDI.

**Table 3:** Caffeine and neuroimaging association with neurodevelopmental impairment.

		Any NDI	
**Predictor**	**OR**	**95% C.I**.	***p*** **value**
Univariate			
Age at first dose	1.02	0.96, 1.08	0.522
Maintenance dose	0.77	0.56, 1.07	0.122
ADCE	0.69	0.50, 0.95	**0.016**
CCE	1.00	0.99, 1.00	0.051
WMI severity	1.37	0.62, 3.03	0.440
GMH/IVH Severity	1.14	0.70, 1.84	0.598
Multivariable^a^			
Age at first dose	1.01	0.95, 1.07	0.831
Maintenance dose	0.73	0.52, 1.04	0.079
ADCE	0.58	0.39, 0.87	**0.009**
CCE	0.99	0.99, 1.00	**0.010**
WMI Severity	1.55	0.66, 3.62	0.312
GMH/IVH Severity	1.13	0.68, 1.88	0.641

*CCE* cumulative caffeine exposure, *ADCE* average daily caffeine exposure, *WMI Severity* white matter injury severity on last brain MRI scan during the neonatal hospitalization, *GMH/IVH Severity* germinal matrix hemorrhage/intraventricular hemorrhage severity on last brain MRI scan during the neonatal hospitalization.

^a^Multivariable models adjusted for gestational age at birth and corrected age at follow-up.

Bold values indicate statistical significance *p* < 0.05.

We conducted sensitivity analyses to address the possibility that the association between caffeine exposure and neurodevelopmental outcome might be explained by worse outcomes specifically in subjects who did not receive any caffeine. We found a similar association of ADCE and CCE with NDI when the subjects who did not receive any caffeine were removed from the cohort (Supplementary Table S2). Thus, higher, more sustained dosing of caffeine throughout the neonatal hospitalization is associated with improved neurodevelopmental performance at 30 months corrected age.

### Characteristics of caffeine dose groups

**Fig. 1:**
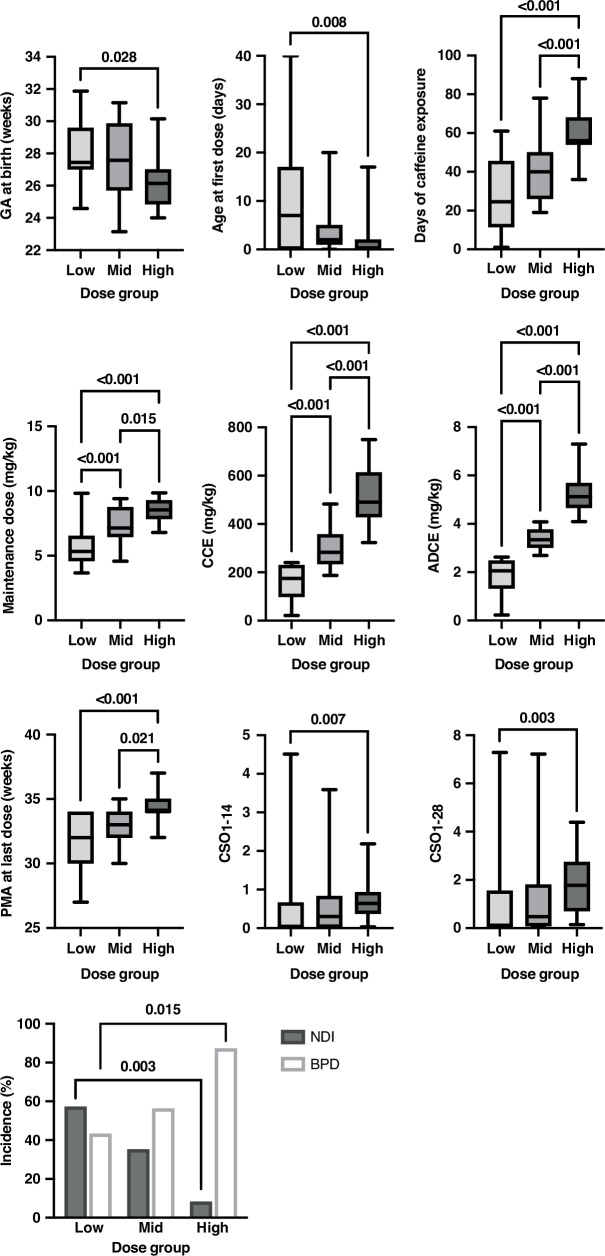
Pairwise comparisons of characteristics and dosing parameters across caffeine dose groups. Continuous variables were compared by one-way ANOVA with post-hoc Tukey’s tests (gestational age [GA] at birth, maintenance dose, cumulative caffeine exposure [CCE], postmenstrual age [PMA] at last dose), Welch’s ANOVA with post-hoc Dunnet’s tests (days of caffeine) or Kruskal-Wallis with post-hoc Dunn’s tests (age at first dose, average daily caffeine exposure [ADCE], cumulative supplemental oxygen during the first 14 days of life [CSO_1–14_] and cumulative supplemental oxygen during the first 28 days of life [CSO_1–28_]). Categorical variables were compared by Fisher’s exact tests followed by Bonferroni correction for pairwise comparisons.

To better understand characteristics and outcomes in subjects with high ADCE, and to identify target caffeine dosing parameters, we ranked the 69 subjects with neurodevelopmental follow-up by ADCE, followed by grouping into tertiles. Characteristics of each dose group (high, mid, and low dose caffeine) were compared (Supplementary Table S3 and Fig. 1). CCE (*p* < 0.001, one-way ANOVA), maintenance dose (*p* < 0.001, one-way ANOVA), age at first dose (*p* = 0.009, Kruskal–Wallis test), and PMA at last dose (*p* < 0.001, one-way ANOVA) differed between the low, mid, and high dose groups (see Fig. 1 for pairwise comparisons). Loading doses did not differ across groups (*p* = 0.122, one-way ANOVA). Subjects in the high-dose group displayed characteristics previously associated with a higher risk of NDI, including a lower GA at birth compared to the low-dose group (*p* = 0.028, one-way ANOVA followed by post hoc Tukey’s test), a higher frequency of BPD compared to the low-dose group (*p* = 0.015, Fisher’s exact test with Bonferroni correction for multiple comparisons) and higher cumulative supplemental oxygen (CSO) during the first 14 days (CSO_1–14_) of life (*p* = 0.007, Kruskal–Wallis followed by post hoc Dunn’s test) and first 28 days (CSO_1–28_) of life (*p* = 0.003, Kruskal–Wallis followed by post hoc Dunn’s test) compared to the low-dose group. Despite these characteristics, subjects in the high-dose caffeine group had a lower frequency of NDI compared to the low-dose group (*p* = 0.003, Fisher’s exact test with Bonferroni correction for multiple comparisons).

### Caffeine dose group association with motor, language and cognitive performance

**Fig. 2:**
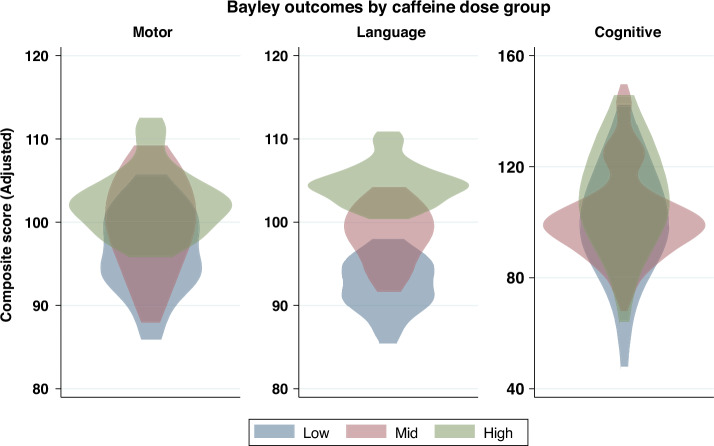
Motor, language and cognitive performance on Bayley-III assessment at 30 months corrected age. Overlaid violin plots show the distribution of composite Bayley scores in each domain in low (blue), mid (red) and high (green) dose caffeine groups. Scores were adjusted for gestational age at birth and cumulative supplemental oxygen during the first 14 days of life using multivariable linear regression.

We used linear regression to test for differences in composite scores on the Bayley-III language, motor, and cognitive subscales between dose groups (Table 4 and Fig. 2). In univariate analyses, high dose caffeine was associated with improved language scores as compared to low dose caffeine (mean difference 11.8, 95% C.I. 2.0, 21.6, *p* = 0.019). We also tested several multivariable models in which GA at birth and severity of respiratory disease (BPD, CSO_1–14_, or CSO_1–28_) were included as covariates, given the differences in these variables observed across caffeine dose groups (Supplementary Table S3). High dose caffeine was associated with improved motor and language scores compared to low dose caffeine in models that included BPD or CSO_1–28_ as covariates. In the model that included CSO_1–14_ as a covariate, motor, language and cognitive scores were all higher in the high dose caffeine group compared to the low dose group (motor mean difference 10.9, 95% C.I. 0.7, 21.0, *p* = 0.036, language mean difference 15.2, 95% C.I. 3.4, 27.0, *p* = 0.012, cognitive mean difference 13.0, 95% C.I. 0.6, 25.4, *p* = 0.040).

**Table 4:** Caffeine dose groups and developmental domain-specific performance.

Predictor	Motor score		Language score		Cognitive score	
	Mean difference (95% CI)	*p* value	Mean difference (95% CI)	*p* value	Mean difference (95% CI)	*p* value
Univariate						
Low dose group	Ref		Ref		Ref	
Mid dose group	5.8 (−2.9, 14.5)	0.190	6.0 (−3.7, 15.8)	0.222	2.4 (−7.9, 12.7)	0.646
High dose group	5.1 (−3.6, 13.8)	0.248	11.8 (2.0, 21.6)	**0.019**	8.9 (−1.4, 19.2)	0.090
Multivariable^a^						
Low dose group	Ref		Ref		Ref	
Mid dose group	7.5 (−1.1, 16.1)	0.086	6.9 (−2.8, 16.6)	0.162	3.2 (−7.1, 13.5)	0.534
High dose group	9.4 (0.0, 18.8)	**0.049**	13.3 (2.6, 24.0)	**0.015**	10.4 (−0.8, 21.8)	0.069
Multivariable^b^						
Low dose group	Ref		Ref		Ref	
Mid dose group	8.3 (−0.7, 17.3)	0.071	7.2 (−3.3, 17.7)	0.174	3.4 (−7.6, 14.4)	0.539
High dose group	10.9 (0.7, 21.0)	**0.036**	15.2 (3.4, 27.0)	**0.012**	13.0 (0.6, 25.4)	**0.040**
Multivariable^c^						
Low dose group	Ref		Ref		Ref	
Mid dose group	8.0 (−1.0, 17.0)	0.080	7.0 (−3.5, 17.5)	0.188	3.0 (−8.1, 14.1)	0.588
High dose group	10.3 (0.3, 20.4)	**0.044**	14.7 (3.1, 26.4)	**0.014**	11.9 (−0.4, 24.2)	0.058

Multivariable linear regression models included GA at birth and:

^a^BPD.

^b^CSO_1–14_.

^c^CSO_1–28_ as covariates.

Bold values indicate statistical significance *p* < 0.05.

The superior performance of the high dose caffeine group in motor, cognitive, and language domains suggests that the neuroprotective effects of caffeine might be optimized at the ADCE of the high dose group. We calculated the length of treatment required for a range of gestational ages at birth to achieve the median ADCE in the high dose group (Table S4). In our calculations, we assumed that patients would receive a loading dose of 20 mg/kg within the first 24 h of life, and we assumed that all infants born at less than 26 weeks would be treated with caffeine through at least 35 weeks PMA, as is the current practice in many institutions.^27^ We found that the goal minimum ADCE was achievable for the full range of gestational ages with a maintenance dose regimen of 10 mg/kg/day, but not 8 mg/kg/day.

## Discussion

In this study, we found that higher cumulative and average daily caffeine exposure in preterm infants were associated with lower odds of NDI at 30 months CA. Motor, language, and cognitive performance on the Bayley-III was superior in neonates in the high dose group compared to subjects in the low dose group, despite an increased rate of BPD and a lower GA at birth in the high dose group. These findings suggest that the neuroprotective and neurorestorative effects of caffeine may ameliorate known risk factors for acquired brain injury and NDI.^18^ We also found that several caffeine dosing parameters previously tested in premature infants, including age at first (loading) dose and maintenance dose, were not associated with NDI in this cohort.^10,28^ We propose that relatively high, sustained caffeine exposures throughout the neonatal hospitalization may be particularly beneficial to the preterm brain. Small differences in timing of caffeine administration may be less critical, while the effect of maintenance dose selection may depend on the length of the dosing period.

The neuroprotective effects of caffeine on the preterm brain have been partially attributed to optimized brain oxygenation resulting from improved respiratory drive and reduced need for ventilatory assistance.^29^ Longer duration and higher overall caffeine exposure may more effectively decrease cumulative exposure to intermittent hypoxemia.^30^ Preclinical and human studies have demonstrated that caffeine also stimulates oligodendrocyte differentiation and enhances myelination,^16,29,31^, and reduces neuronal death and microgliosis.^32,33^ In the current study, we did not observe an association between caffeine exposure and rate or severity of acquired brain injury of prematurity, including WMI, GMH/IVH, and cerebellar hemorrhage, after controlling for potential confounders. It is possible that caffeine may not prevent brain injury and may instead promote recovery after injury. However, our power to detect differences in rates of brain injury was limited due to an overall low number of subjects with severe brain injury.^34^ Low rates of brain injury and of NDI in our cohort may be partially attributable to selection bias, as the most critically ill preterm neonates in the intensive care nursery, including those with severe brain injury, may not have been stable enough for transport to brain MRI during the timeframe of the study. Those patients would have been excluded from the study, resulting in apparent rates of acquired brain injury and NDI that are lower than the general preterm population. Patients who received higher caffeine exposures tended to also have more risk factors for acquired brain injuries, such as BPD, which further hindered our ability to assess for an association between caffeine exposure and brain injury.^18,26^ We also note that brain MRIs in this study were performed at a median age of 36.1 weeks PMA (Table 1). Caffeine administration was ongoing in some patients at the time of brain MRI. Randomized controlled trials that include later-interval MRIs, detailed qualitative scales of acquired brain injury severity, and advanced imaging metrics of brain microstructure and myelination would enable a more robust analysis of the effects of caffeine on brain structure and recovery after injury.^31,35,36^

When testing for an association between caffeine dose group and neurodevelopmental performance, we examined several different potential confounders reflective of severity of respiratory disease. The strongest association between caffeine dose group and neurodevelopmental outcomes was seen after adjusting for CSO_1–14_ in a multivariable analysis. CSO was previously demonstrated to be independently associated with BPD or death at 36 weeks PMA, with 1-year respiratory morbidity, and with neurodevelopmental performance at 30 months CA in this same cohort, with a predictive accuracy that plateaued at 14 days.^18,21,37^ It is unclear whether CSO is simply a biomarker for respiratory illness severity or if interventions to reduce CSO could be implemented alongside caffeine therapy in preterm infants to further optimize neurodevelopmental outcomes.

An important limitation of this study was the loss to follow-up rate of 35%, which may have introduced additional selection bias in our analyses, although baseline characteristics and imaging findings were not different between patients with and without follow-up. The observational design was also a significant limitation. Caffeine exposure was allocated based on clinical guidelines, and subjects with higher caffeine exposures tended to be sicker and to have an earlier GA at birth. Furthermore, in current clinical practice, maintenance doses of caffeine are only increased in the setting of ongoing symptoms of apnea of prematurity. The resulting confounding by indication would tend to bias this study towards the null. Nearly all infants in this study received a loading dose of 20 mg/kg, which precluded assessment of the effects of different loading doses. Additionally, no patients received maintenance dosing higher than 10 mg/kg/day, and it remains unclear whether higher maintenance doses could further enhance the neuroprotective effects of caffeine. A recent Phase IIb trial demonstrated that maintenance doses of 20 mg/kg/day improved short-term respiratory parameters as compared to lower maintenance doses or placebo.^30^ The long-term neurodevelopmental outcomes of the patients have not yet been reported and may shed light onto how to further optimize the neuroprotective effects of caffeine. Finally, neurodevelopmental outcomes in this study were based on the Bayley-III examination, which has been reported to under-identify children at risk of future neurodevelopmental impairment.^38,39^

In summary, higher cumulative and average daily caffeine exposure are associated with improved neurodevelopmental outcomes in preterm infants. We identified dosing regimens that would deliver the median ADCE (5.1 mg/kg) of the high dose group to infants born at a range of gestational ages. The goal minimum ADCE is achievable for all ages with a caffeine bolus of 20 mg/kg followed by maintenance dosing of 10 mg/kg/day until 34–36 weeks PMA. Length of treatment course is dependent on GA at birth. Neonatal intensive care units could consider updating clinical guidelines to target specific caffeine exposures in preterm infants. However, the optimal caffeine dosing regimen for neuroprotection remains unclear. Future prospective studies that include higher maintenance doses, follow-up neuroimaging, and neurodevelopmental testing at older ages would further clarify the impact of caffeine exposure in this population. Finally, while caffeine has an important role to play in preterm neuroprotection, there remains a critical need for novel prevention strategies and targeted treatments for acquired brain injury in this vulnerable population.^40–43^

## Supplementary information

Supplementary Data

## Data Availability

The data generated during the current study are available from the contact authors on reasonable request.
