# Supplementary material for: Cumulative caffeine exposure predicts neurodevelopmental outcomes in premature infants
Source: Pediatr Res. 2025 Sep 20;99(7):2605–13. doi: 10.1038/s41390-025-04387-1 (PMC13485691; doi:10.1038/s41390-025-04387-1)
Supplement: Supplementary file 1 [file 41390_2025_4387_MOESM1_ESM.pdf]

Ostrem et al Supplementary Data

Table S1 Characteristics of patients with and without neurodevelopmental follow-up

Neonatal characteristics	Study Cohort (N=106)	Follow-up not available (n=37)	Follow-up available (n=69)	p-value*
GA at birth, mean wks (SD)	27.7 (2.2)	28.2 (2.1)	27.5 (2.2)	0.099
Birthweight, median grams (IQR)	980 (750, 1255)	935 (765, 1401)	990 (730, 1175)	0.520
Male, n (%)	54 (51)	19 (51)	35 (51)	1.000
Prenatal steroids, n (%)	82 (77)	28 (77)	54 (78)	0.810
Maternal chorioamnionitis, n (%)	11 (10)	4 (11)	7 (10)	1.000
PDA, n (%)	60 (57)	16 (43)	44 (64)	0.064
Necrotizing enterocolitis, n (%)	6 (6)	1 (3)	5 (7)	0.662
BPD, n (%)	61 (57)	17 (46)	43 (62)	0.150
Received caffeine, n (%)	97 (92)	33 (89)	64 (93)	0.716
Age at first caffeine dose, median days (IQR)	1 (0,5)	1 (0, 4)	1.5 (0, 6.5)	0.383
Loading caffeine dose, median mg/kg (IQR)	20.2 (20.0. 21.2)	20.1 (19.8, 21.1)	20.3 (20.0, 21.2)	0.456
Maintenance dose, median mg/kg (IQR)	7.6 (6.3, 8.7)	7.5 (5.5, 8.4)	7.6 (6.3, 8.8)	0.743
Average daily caffeine exposure (ADCE), mean mg/kg (SD)	3.3 (1.9)	3.2 (2.1)	3.4 (1.8)	0.652
Cumulative caffeine exposure (CCE), mean mg/kg (SD)	301.3 (196.4)	286.3 (215.6)	309.3 (186.5)	0.567
Postmenstrual age at MRI, median weeks (IQR)	36.1 (34.9, 37.9)	36.7 (35.4, 38.9)	36.1 (34.6, 37.7)	0.162
WMI on MRI, available N	N=89	N=30	N=59	0.317
None, n (%)	64 (72)	20 (67)	44 (75)	
Mild, n (%)	16 (18)	5 (17)	11 (19)	
Moderate, n (%)	5 (6)	2 (7)	3 (5)	
Severe, n (%)	4 (4)	3 (10)	1 (2)	
IVH/GMH on MRI, available N	N=101	N=33	N=68	0.951
None, n (%)	69 (68)	23 (70)	46 (68)	
Grade 1, n (%)	8 (8)	3 (9)	5 (7)	
Grade 2, n (%)	16 (16)	3 (9)	13 (19)	
Grade 3, n (%)	4 (4)	1 (3)	3 (4)	
PVHI, n (%)	4 (4)	3 (9)	1 (1)	
Cerebellar hemorrhage, n out of available N (%)	30/101 (30)	7/33 (21)	23/68 (34)	0.248
Death before discharge, n (%)	2 (2)	2 (5)	0 (0)	0.120

*SD, standard deviation. IQR, interquartile range. WMI, white matter injury severity on last brain MRI scan during the neonatal hospitalization. IVH/GMH, intraventricular hemorrhage or germinal matrix hemorrhage severity on last brain MRI scan during the neonatal hospitalization. PVHI, periventricular hemorrhagic infarction. Available N are shown for neuroimaging findings, as motion artifact precluded full characterization of all MRI features in all scans. *comparisons made between subjects with neurodevelopmental follow-up at 30 months CA, and subjects without available neurodevelopmental follow-up. Categorical variables were compared with Fisher's exact tests, ordinal variables were compared with Wilcoxon rank-sum tests, and continuous variables were compared with Student's t-tests or Wilcoxon rank-sum tests.*

Table S2. Sensitivity analysis of caffeine and neuroimaging association with neurodevelopmental impairment

	Any NDI		
**Predictor**	**OR**	**95% C.I.**	**p-value**
Univariate			
Age at First Dose	1.02	0.96, 1.08	0.522
Maintenance Dose	0.77	0.56, 1.07	0.117
ADCE	0.68	0.47, 1.00	**0.040**
CCE	1.00	0.99, 1.00	0.127
WMI Severity	1.46	0.40, 5.38	0.574
GMH/IVH Severity	1.15	0.23, 0.83	0.802
Multivariable*			
Age at First Dose	1.01	0.95, 1.07	0.831
Maintenance Dose	0.73	0.52, 1.04	0.079
ADCE	0.58	0.37, 0.92	**0.020**
CCE	0.99	0.99, 1.00	**0.022**
WMI Severity	2.21	0.50, 9.75	0.295
GMH/IVH Severity	1.08	0.34, 3.50	0.145

*Logistic regression was used to determine the association of caffeine dosing parameters and neuroimaging findings with neurodevelopmental impairment (NDI), defined as a score of ≤85 on motor, language, and/or cognitive subscales of the Bayley-III. Patients who received no caffeine (N=5) were removed from the analysis. CCE, cumulative caffeine exposure; ADCE, average daily caffeine exposure; WMI Severity, white matter injury severity on last brain MRI scan during the neonatal hospitalization; GMH/IVH Severity, germinal matrix hemorrhage/ intraventricular hemorrhage severity on last brain MRI scan during the neonatal hospitalization.*Multivariable models adjusted for gestational age at birth and corrected age at follow-up.*

Table S3. Characteristics of caffeine dose groups

	Caffeine Dose Group			
**Characteristic**	**Low (n=23)**	**Mid (n=23)**	**High (n=23)**	**p-value**
GA at birth, mean wks (SD)	28.5 (2.1)	27.7 (2.3)	26.3 (1.7)	**0.017**[a]
BPD, n (%)	10 (43)	13 (56)	20 (87)	**0.007**[d]
$CSO_{1\text{-}14}$, median (IQR)	0.04 (0, 0.37)	0.30 (0.01, 0.83)	0.64 (0.39, 0.91)	**0.009**[c]
$CSO_{1\text{-}28}$, median (IQR)	0.12 (0, 1.42)	0.47 (0.08, 1.80)	1.77 (0.73, 2.71)	**0.004**[c]
Age at first caffeine dose, median days (IQR)	7 (0, 16)	2 (1, 5)	0 (0, 2)	**0.009**[c]
Loading dose, mean mg/kg (SD)	19.6 (3.4)	21.2 (1.9)	20.6 (2.1)	0.122[a]
Maintenance dose, mean mg/kg (SD)	5.8 (1.6)	7.4 (1.4)	8.5 (1.4)	**<0.001**[a]
ADCE, median mg/kg (IQR)	1.9 (0.2, 2.0)	3.3 (3.0, 3.8)	5.1 (4.7, 5.7)	**<0.001**[c]
CCE, mean mg/kg (SD)	123.6 (93.0)	294.4 (82.1)	510.1 (116.7)	**<0.001**[a]
Days of caffeine, mean (SD)	27 (18)	39 (14)	59 (13)	**<0.001**[b]
PMA at last dose, mean weeks (SD)	31.5 (2.2)	32.9 (1.4)	34.3 (1.1)	**<0.001**[a]
NDI, n (%)	13 (57)	8 (35)	2 (8)	**0.002**[d]

GA, gestational age. BPD, bronchopulmonary dysplasia. SD, standard deviation. IQR, interquartile range. $CSO_{1\text{-}14}$, cumulative supplemental oxygen during the first 14 days of life. $CSO_{1\text{-}28}$, cumulative supplemental oxygen during the first 28 days of life. ADCE, average daily caffeine exposure. CCE, cumulative caffeine exposure. PMA, postmenstrual age. NDI, neurodevelopmental impairment. Continuous variables compared by [a]one-way ANOVA, [b]Welch's ANOVA or [c]Kruskal-Wallis tests. [d]Categorical variables compared by Fisher's exact tests.

Table S4. Length of caffeine treatment course needed to achieve the average daily caffeine exposure in the high-dose group.

GA at Birth (weeks)	Maintenance Dose (mg/kg)	Min. weeks of treatment	Min. PMA at last dose[a]	ADCE (mg/kg)	CCE (mg/kg)
23 and 0/7 to	8	11	35 weeks	5.7	636
23 and 6/7	10	11	35 weeks	7.0	790
24 and 0/7 to	8	11	36 weeks	5.3	636
24 and 6/7	10	10	35 weeks	6.8	720
25 and 0/7 to	8	12	37 weeks	5.5	692
25 and 6/7	10	9	35 weeks	6.6	650
26 and 0/7 to	8	*	*	*	*
26 and 6/7	10	7	34 weeks	5.5	510
27 and 0/7 to	8	*	*	*	*
27 and 6/7	10	6	34 weeks	5.2	440
28 and 0/7 to	8	*	*	*	*
28 and 6/7	10	6	35 weeks	5.6	440
29 and 0/7 to	8	*	*	*	*
29 and 6/7	10	5	35 weeks	5.2	370
30 and 0/7 to	8	*	*	*	*
30 and 6/7	10	5	36 weeks	5.8	370
31 and 0/7 to	8	*	*	*	*
31 and 6/7	10	4	36 weeks	5.3	300

*Length of treatment course needed to achieve specific caffeine exposures over the neonatal hospitalization. We assumed a loading dose of 20 mg/kg on the first day of life. Target average daily caffeine exposure (ADCE) is ≥5.1 mg/kg, and target postmenstrual age (PMA) at last dose is at least 34 weeks for all gestational ages. [a]Current guidelines (minimum ≥35 weeks PMA) were used to determine age at last dose for neonates born at < 26 weeks. *Maintenance dosing of 8 mg/kg could not provide the target ADCE for gestational ages equal to or greater than 26 weeks at birth without continuing dosing beyond 37 weeks PMA, which could require dosing beyond hospital discharge. CCE, cumulative caffeine exposure.*

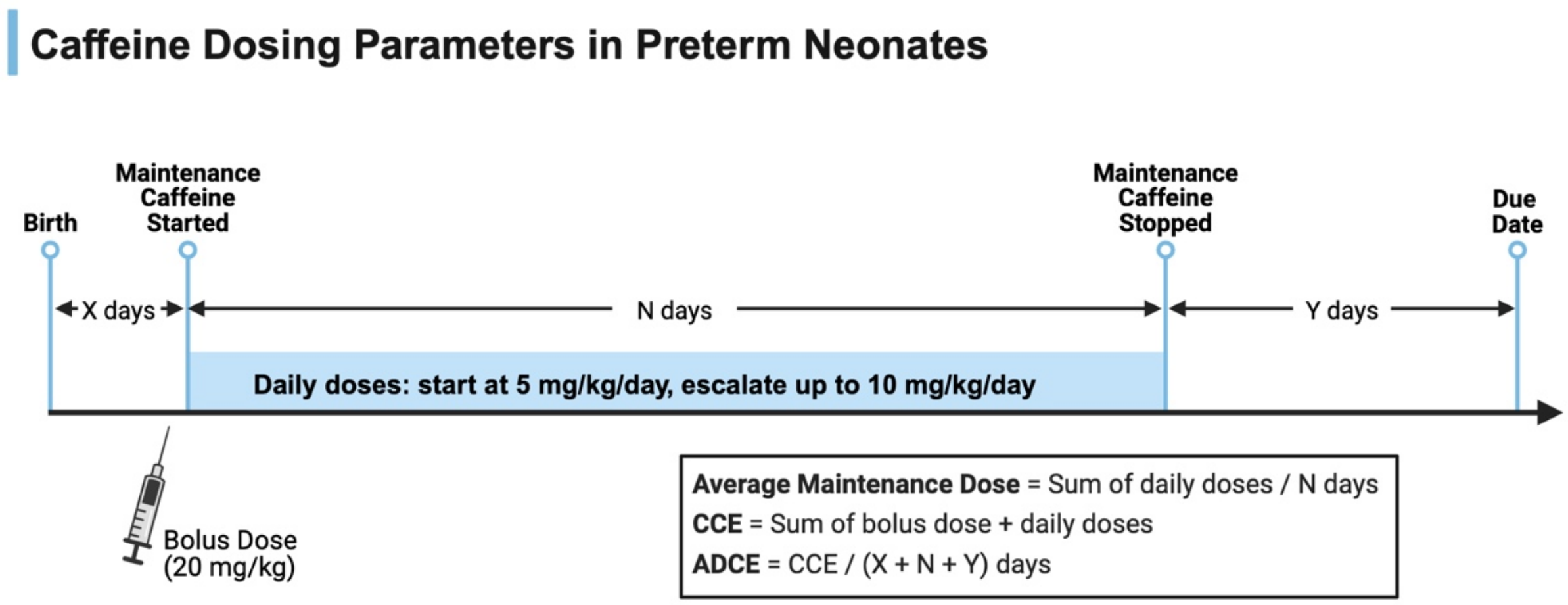

Figure S1. Caffeine Dosing Parameters in Preterm Neonates. Participants in this study who received caffeine were typically given a 20 mg/kg bolus dose, followed by maintenance dosing of between 5-10 mg/kg/day based on respiratory indications. CCE, cumulative caffeine exposure. ADCE, Average daily caffeine exposure. Created with biorender.com.
